# Profiling transcription initiation in human aged brain using deep-CAGE

**DOI:** 10.1186/1471-2105-12-S11-A8

**Published:** 2011-11-21

**Authors:** Margherita Francescatto, Luba Pardo, Patrizia Rizzu, Morana Vitezic, Javier Simón-Sánchez, Hazuki Takahashi, Carsten Daub, Piero Carninci, Peter Heutink

**Affiliations:** 1 Department of Clinical Genetics, Section Medical Genomics, VU Medical Center, Amsterdam, The Netherlands; 2 GABBA Program, Instituto de Ciências Biomédicas Abel Salazar, UP, Porto, Portugal; 3 RIKEN Omics Science Center, RIKEN Yokohama Institute, Yokohama, Japan; 4 Department of Cell and Molecular Biology (CMB), Karolinska Institute, Stockholm, Sweden

## Introduction

The genome sequencing projects completed in recent years revealed that the number of protein-coding genes does not change appreciably with increasing complexity of the organisms, and it is now generally accepted that this divergence is largely due to variation at the regulatory level. Mechanisms such as alternative splicing, alternative promoters and antisense transcription allow to both obtain a high number of transcripts from a relatively small number of genes and to fine tune isoforms expression in a cell-specific or developmentally-restricted manner. It is likely that the extensive use of such mechanisms plays a pivotal role in development, adult function and ageing of complex tissues like brain.

The aim of this study was to characterize transcription start sites (TSSs) in different areas of human aged brain and correlate expression with methylation and structural genomic variation. Since its ability to profile TSSs at high resolution and at a genome wide level, we used Cap Analysis of Gene Expression (CAGE) combined with high-throughput sequencing (deep-CAGE) to collect exact TSSs and their expression levels. We present here our findings on alternative promoters and antisense transcription. Post-mortem tissue from 5 different brain regions was collected from 5 human donors and used to prepare 25 libraries.

## Results

On average 2 million CAGE tags for each sample were sequenced. Mapping, expression normalization and clustering of the tags were carried out using automated pipelines. Core promoters were defined by merging tags within 300 base pairs of each other on the same strand. We found 22023 promoters, 50% of which mapped to either the promoter region or the 5' UTR of RefSeq transcripts. Ca. 32% of the genes expressed use alternative promoters. Ca. 15% of the promoters found were either part of a bi-directionally transcribed pair or antisense to an annotated RefSeq gene. A promoter was considered preferentially expressed (PEP) in one of the regions if at least 50% of its expression was derived from that region. Around 30% of the alternative promoters were PEP in one of the regions. In 8% of the bi-directional pairs identified, at least one of the members was PEP while 35% of the antisense promoters was.

## Conclusions

This study confirms deep-CAGE as a suitable approach to characterize mechanisms involved in the regulation of gene expression, such as alternative promoter usage and antisense transcription, even in the challenging context given by the use of post-mortem tissue from aged human brain.

